# Aqua­[2,2′-(propane-1,3-di­yl)bis­(5-car­boxy-1*H*-imidazole-4-carboxyl­ato)-κ^4^ *N* ^3^,*O* ^4^:*N* ^3′^,*O* ^4′^](pyridine-κ*N*)cobalt(II)–4,4′-bipyridine (1/1)

**DOI:** 10.1107/S1600536812029856

**Published:** 2012-07-25

**Authors:** Wei Liu, Xia Li

**Affiliations:** a Department of Chemistry and Chemical Engineering, Henan University of Urban Construction, Pingdingshan, Henan 467044, People’s Republic of China

## Abstract

In the title compound, [Co(C_13_H_10_N_4_O_8_)(C_5_H_5_N)(H_2_O)]·C_10_H_8_N_2_, the asymmetric unit comprises half a Co^II^ complex located on a mirror plane and half a cocrystallized mol­ecule of 4,4′-bipyridine located on an inversion center. The Co^II^ ion is six coordinate, with distorted octa­hedral geometry, ligated by two N atoms and two O atoms from a 2,2′-(propane-1,3-di­yl)bis­(5-carboxy-1*H*-imidazole-4-carboxyl­ate) dianion, one N atom from a pyridine mol­ecule and one coordinating water mol­ecule. The Co—O bond lengths range from 2.076 (2) to 2.1441 (15) Å, while the Co—N bond lengths are 2.138 (3) and 2.1515 (17) Å. A two-dimensional network of N—H⋯O and O—H⋯N hydrogen bonds stabilizes the crystal packing. There are π–π inter­actions between the bipyridine and imidazole rings [centroid–centroid distance = 3.7694 (4) Å]. The propane-1,3-diyl group is disordered over two conformations, with refined occupancies of 0.755 (8) and 0.245 (8).

## Related literature

For complexes based on substituted 4,5-imidazole­dicarb­oxy­lic acids, see: Zhu *et al.* (2010 ▶, 2011 ▶); Lu *et al.* (2010 ▶); Song *et al.* (2010 ▶); Zhang *et al.* (2010 ▶); Wang *et al.* (2008 ▶); Feng *et al.* (2010 ▶); Liu *et al.* (2010 ▶); Zheng *et al.* (2011 ▶); Li *et al.* (2009 ▶, 2010 ▶).
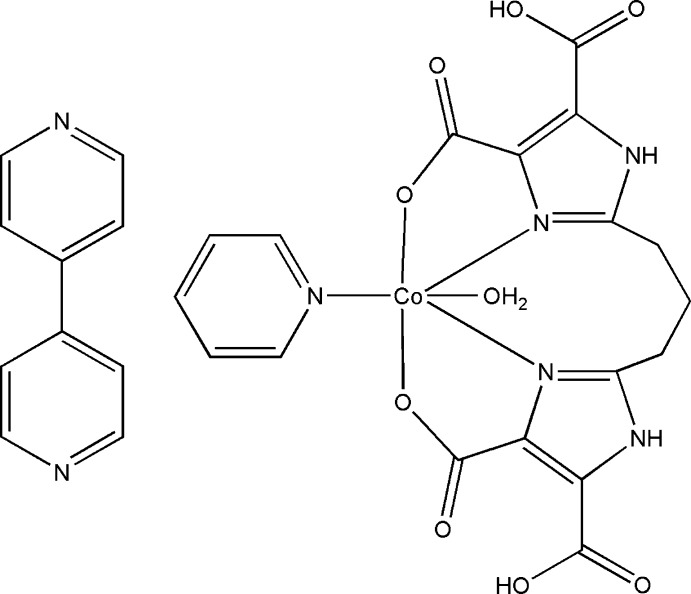


## Experimental

### 

#### Crystal data

[Co(C_13_H_10_N_4_O_8_)(C_5_H_5_N)(H_2_O)]·C_10_H_8_N_2_
*M* *_r_* = 661.47
Monoclinic, 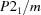

*a* = 7.9733 (10) Å
*b* = 20.738 (3) Å
*c* = 8.2987 (11) Å
β = 91.350 (2)°
*V* = 1371.8 (3) Å^3^
*Z* = 2
Mo *K*α radiation
μ = 0.70 mm^−1^
*T* = 296 K
0.22 × 0.18 × 0.11 mm

#### Data collection

Bruker SMART CCD area-detector diffractometer
Absorption correction: multi-scan (*SADABS*; Sheldrick, 2001 ▶) *T*_min_ = 0.862, *T*_max_ = 0.927
11480 measured reflections
3443 independent reflections
2633 reflections with *I* > 2σ(*I*)
*R*_int_ = 0.033

#### Refinement

*R*[*F* ^2^ > 2σ(*F* ^2^)] = 0.043
*wR*(*F* ^2^) = 0.105
*S* = 1.05
3443 reflections
219 parameters
1 restraint
H-atom parameters constrained
Δρ_max_ = 0.43 e Å^−3^
Δρ_min_ = −0.34 e Å^−3^

### 

Data collection: *SMART* (Bruker, 2001 ▶); cell refinement: *SAINT* (Bruker, 2001 ▶); data reduction: *SAINT*; program(s) used to solve structure: *SHELXS97* (Sheldrick, 2008 ▶); program(s) used to refine structure: *SHELXL97* (Sheldrick, 2008 ▶); molecular graphics: *SHELXTL* (Sheldrick, 2008 ▶); software used to prepare material for publication: *SHELXTL*.

## Supplementary Material

Crystal structure: contains datablock(s) I, global. DOI: 10.1107/S1600536812029856/pk2422sup1.cif

Structure factors: contains datablock(s) I. DOI: 10.1107/S1600536812029856/pk2422Isup2.hkl

Additional supplementary materials:  crystallographic information; 3D view; checkCIF report

## Figures and Tables

**Table 1:** Hydrogen-bond geometry (Å, °)

*D*—H⋯*A*	*D*—H	H⋯*A*	*D*⋯*A*	*D*—H⋯*A*
O3—H3⋯O2	0.82	1.65	2.473 (2)	177
O5—H1*W*⋯N4^i^	0.82	1.95	2.727 (3)	157
N2—H2⋯O4^ii^	0.86	1.93	2.763 (3)	162

Symmetry codes: (i) 
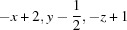
; (ii) 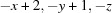.

**Fig. 1.**
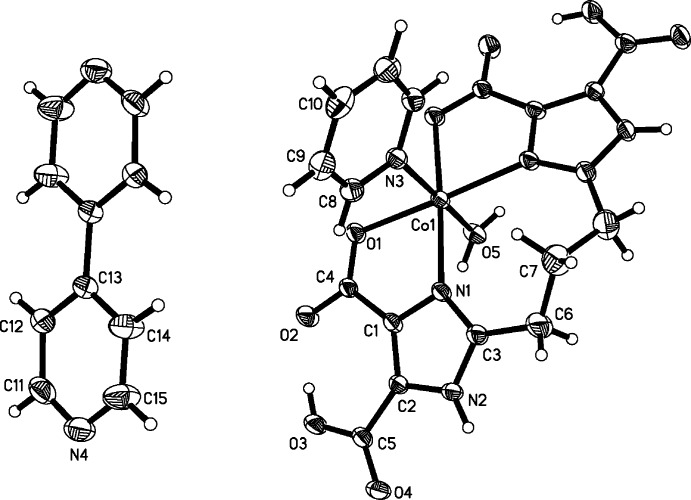
The molecular structure of the title compound, showing 30% probability displacement ellipsoids. Unlabelled atoms are related to labelled atoms by the symmetry operations (x, -y+1/2, z for CoII complex; 1-x, 1-y, 1-z for 4,4-bipy).

## supplementary crystallographic information

### Comment

It is well known that aromatic polycarboxylates, especially the N-heterocyclic carboxylates, are excellent candidates for preparing novel MOFs, because of their versatile coordination modes and potential hydrogen-bonding donors and acceptors. Recently, 4,5-imidazoledicarboxylic acid (Zhu *et al.*, 2010; Lu *et al.*, 2010) and its 2-position substituent derivatives, such as 2-methyl-1*H*-imidazole-4,5-dicarboxylic acid (Song *et al.*, 2010), 2-ethyl-1*H*-imidazole-4,5-dicarboxylic acid (Zhang *et al.*, 2010; Wang *et al.*, 2008), 2-propyl-1*H*-imidazole-4,5-dicarboxylic acid (Feng *et al.*, 2010; Liu *et al.*, 2010), 2-(hydroxymethyl)-1*H*-imidazole-4,5-dicarboxylic acid (Zheng *et al.*, 2011), 2-phenyl-1*H*-imidazole-4,5-dicarboxylic acid (Zhu *et al.*, 2011) and 2-pyridyl-1*H*-imidazole-4,5-dicarboxylic acid (Li *et al.*, 2009; Li *et al.*, 2010) have attracted great attention in the field of coordination chemistry. Now, our group has strong interest in adopting another imidazole dicarboxylate ligand, 1,3-bis-(1*H*-imidazole-4,5-dicarboxylate acid) propane to prepare various coordination compounds.

As shown in Fig. 1, the molecule is a discrete neutral monomer, in which the asymmetric unit comprises half a CoII complex located on a mirror plane and half a co-crystallized molecule of 4,4'-bipyridine located on an inversion center. The Co^II^ ion is six coordinate and has a distorted octahedral geometry. It is ligated by two nitrogen atoms and two oxygen atoms from a 1,3-bis-(1*H*-imidazole-4,5-dicarboxylate) propane dianion, one nitrogen atom from a pyridine molecule and one oxygen atom from a coordinated water molecule. The Co—O distances range from 2.076 (2) to 2.1441 (15) Å, while Co—N distances are 2.138 (3) and 2.1515 (17) Å, respectively. A two-dimensional network of N—H···O and O—H···N hydrogen bonds help to stabilize the crystal packing. Aromatic π-π interactions between bipyridine rings and imidazole rings [centroid—centroid distance = 3.7694 (4) Å] are also observed.

### Experimental

A mixture of cobalt chloride hexahydrate (0.0238 g, 0.1 mmol), 1,3-bis-(1*H*-imidazole-4,5-dicarboxylate acid) propane (0.0352 g, 0.1 mmol), 4,4'-bipyridine (0.0198 g, 0.1 mmol), pyridine (0.8 ml) and H_2_O (10 ml) was sealed in a Teflon-lined stainless autoclave and heated at 413 K for 3 days. The bomb was allowed to cool to room temperature gradually and red prismatic crystals were obtained.

### Refinement

H atoms attached to N and O atoms were located in a difference Fourier maps and refined as riding in their as-found relative positions, with *U*_iso_(H) = 1.5*U*_eq_(O, N). Other H atoms were positioned geometrically with C—H = 0.93 and 0.97 Å for aromatic and methylene H, and constrained to ride on their parent atoms with *U*_iso_(H) = 1.2*U*_eq_(C).

### Crystal data

**Table:**

[Co(C_13_H_10_N_4_O_8_)(C_5_H_5_N)(H_2_O)]·C_10_H_8_N_2_	*F*(000) = 680
*M**_r_* = 661.47	*D*_x_ = 1.601 Mg m^−^^3^
Monoclinic, *P*2_1_/*m*	Mo *K*α radiation, λ = 0.71073 Å
Hall symbol: -P 2yb	Cell parameters from 2701 reflections
*a* = 7.9733 (10) Å	θ = 2.6–28.3°
*b* = 20.738 (3) Å	µ = 0.70 mm^−^^1^
*c* = 8.2987 (11) Å	*T* = 296 K
β = 91.350 (2)°	Prismatic, red
*V* = 1371.8 (3) Å^3^	0.22 × 0.18 × 0.11 mm
*Z* = 2	

### Data collection

**Table:**

Bruker SMART CCD area-detector diffractometer	3443 independent reflections
Radiation source: fine-focus sealed tube	2633 reflections with *I* > 2σ(*I*)
Graphite monochromator	*R*_int_ = 0.033
φ and ω scans	θ_max_ = 28.3°, θ_min_ = 2.6°
Absorption correction: multi-scan (*SADABS*; Sheldrick, 2001)	*h* = −10→10
*T*_min_ = 0.862, *T*_max_ = 0.927	*k* = −27→27
11480 measured reflections	*l* = −11→10

### Refinement

**Table:**

Refinement on *F*^2^	Primary atom site location: structure-invariant direct methods
Least-squares matrix: full	Secondary atom site location: difference Fourier map
*R*[*F*^2^ > 2σ(*F*^2^)] = 0.043	Hydrogen site location: inferred from neighbouring sites
*wR*(*F*^2^) = 0.105	H-atom parameters constrained
*S* = 1.05	*w* = 1/[σ^2^(*F*_o_^2^) + (0.0442*P*)^2^ + 0.5516*P*] where *P* = (*F*_o_^2^ + 2*F*_c_^2^)/3
3443 reflections	(Δ/σ)_max_ < 0.001
219 parameters	Δρ_max_ = 0.43 e Å^−^^3^
1 restraint	Δρ_min_ = −0.34 e Å^−^^3^

### Special details

**Table:**

Geometry. All e.s.d.'s (except the e.s.d. in the dihedral angle between two l.s. planes) are estimated using the full covariance matrix. The cell e.s.d.'s are taken into account individually in the estimation of e.s.d.'s in distances, angles and torsion angles; correlations between e.s.d.'s in cell parameters are only used when they are defined by crystal symmetry. An approximate (isotropic) treatment of cell e.s.d.'s is used for estimating e.s.d.'s involving l.s. planes.
Refinement. Refinement of *F*^2^ against ALL reflections. The weighted *R*-factor *wR* and goodness of fit *S* are based on *F*^2^, conventional *R*-factors *R* are based on *F*, with *F* set to zero for negative *F*^2^. The threshold expression of *F*^2^ > σ(*F*^2^) is used only for calculating *R*-factors(gt) *etc*. and is not relevant to the choice of reflections for refinement. *R*-factors based on *F*^2^ are statistically about twice as large as those based on *F*, and *R*- factors based on ALL data will be even larger.

### Fractional atomic coordinates and isotropic or equivalent isotropic displacement parameters (Å^2^)

**Table:**

	*x*	*y*	*z*	*U*_iso_*/*U*_eq_	Occ. (<1)
Co1	1.06603 (5)	0.2500	0.45178 (5)	0.03122 (13)	
O1	1.1510 (2)	0.32701 (7)	0.60553 (18)	0.0383 (4)	
O2	1.2252 (2)	0.43073 (8)	0.6011 (2)	0.0522 (5)	
O3	1.2037 (3)	0.52310 (8)	0.4133 (2)	0.0529 (5)	
H3	1.2128	0.4918	0.4734	0.079*	
O4	1.1130 (3)	0.54174 (8)	0.1645 (2)	0.0529 (5)	
O5	1.3034 (3)	0.2500	0.3543 (3)	0.0427 (6)	
H1W	1.3294	0.2130	0.3301	0.064*	0.50
N1	1.0110 (2)	0.33325 (8)	0.3051 (2)	0.0333 (4)	
N2	0.9918 (2)	0.41425 (8)	0.1356 (2)	0.0376 (4)	
H2	0.9680	0.4358	0.0494	0.045*	
N3	0.8291 (3)	0.2500	0.5671 (3)	0.0362 (6)	
N4	0.5482 (3)	0.64535 (11)	0.7813 (3)	0.0581 (6)	
C1	1.0812 (3)	0.38718 (10)	0.3751 (3)	0.0323 (5)	
C2	1.0707 (3)	0.43808 (10)	0.2707 (3)	0.0339 (5)	
C3	0.9574 (3)	0.35140 (10)	0.1597 (3)	0.0391 (5)	
C4	1.1577 (3)	0.38108 (10)	0.5396 (3)	0.0352 (5)	
C5	1.1307 (3)	0.50565 (11)	0.2790 (3)	0.0389 (5)	
C6	0.8845 (10)	0.3117 (3)	0.0277 (8)	0.077 (2)	0.755 (8)
H6A	0.9736	0.2990	−0.0432	0.092*	0.755 (8)
H6B	0.8060	0.3380	−0.0347	0.092*	0.755 (8)
C7	0.7919 (6)	0.2500	0.0846 (6)	0.0417 (13)	0.755 (8)
H7A	0.6779	0.2500	0.0411	0.050*	0.755 (8)
H7B	0.7867	0.2500	0.2012	0.050*	0.755 (8)
C8	0.7494 (3)	0.30506 (12)	0.6012 (3)	0.0432 (6)	
H8	0.8032	0.3439	0.5807	0.052*	
C9	0.5915 (3)	0.30676 (14)	0.6651 (3)	0.0531 (7)	
H9	0.5404	0.3460	0.6869	0.064*	
C10	0.5108 (5)	0.2500	0.6959 (5)	0.0564 (10)	
H10	0.4033	0.2500	0.7371	0.068*	
C11	0.6117 (4)	0.63819 (13)	0.9295 (4)	0.0565 (7)	
H11	0.6701	0.6727	0.9751	0.068*	
C12	0.5967 (3)	0.58259 (12)	1.0209 (3)	0.0467 (6)	
H12	0.6442	0.5802	1.1241	0.056*	
C13	0.5101 (3)	0.53088 (11)	0.9558 (3)	0.0378 (5)	
C14	0.4429 (4)	0.53878 (13)	0.8010 (4)	0.0604 (8)	
H14	0.3829	0.5054	0.7520	0.072*	
C15	0.4649 (4)	0.59541 (15)	0.7211 (4)	0.0681 (9)	
H15	0.4183	0.5992	0.6177	0.082*	
C7'	0.9726 (4)	0.25000 (15)	−0.0032 (4)	0.048 (4)*	0.245 (8)
H7'1	0.9962	0.2500	−0.1173	0.057*	0.245 (8)
H7'2	1.0762	0.2500	0.0604	0.057*	0.245 (8)
C6'	0.841 (2)	0.3086 (7)	0.049 (2)	0.034 (3)*	0.245 (8)
H6'1	0.8001	0.3323	−0.0446	0.041*	0.245 (8)
H6'2	0.7470	0.2917	0.1074	0.041*	0.245 (8)

### Atomic displacement parameters (Å^2^)

**Table:**

	*U*^11^	*U*^22^	*U*^33^	*U*^12^	*U*^13^	*U*^23^
Co1	0.0391 (2)	0.0229 (2)	0.0313 (2)	0.000	−0.00719 (16)	0.000
O1	0.0514 (10)	0.0286 (8)	0.0344 (9)	−0.0005 (7)	−0.0105 (7)	−0.0007 (7)
O2	0.0784 (13)	0.0338 (9)	0.0434 (10)	−0.0142 (8)	−0.0229 (9)	−0.0026 (7)
O3	0.0842 (14)	0.0277 (9)	0.0458 (11)	−0.0100 (8)	−0.0171 (9)	−0.0005 (7)
O4	0.0819 (13)	0.0294 (9)	0.0466 (11)	−0.0079 (8)	−0.0133 (9)	0.0070 (8)
O5	0.0451 (13)	0.0284 (11)	0.0549 (15)	0.000	0.0038 (11)	0.000
N1	0.0417 (10)	0.0235 (8)	0.0342 (10)	−0.0004 (7)	−0.0091 (8)	−0.0011 (7)
N2	0.0518 (11)	0.0257 (9)	0.0349 (10)	−0.0025 (8)	−0.0109 (8)	0.0041 (8)
N3	0.0409 (15)	0.0319 (14)	0.0356 (15)	0.000	−0.0052 (11)	0.000
N4	0.0629 (15)	0.0422 (13)	0.0696 (17)	0.0037 (11)	0.0090 (12)	0.0145 (11)
C1	0.0381 (11)	0.0237 (10)	0.0349 (12)	0.0008 (9)	−0.0056 (9)	−0.0030 (8)
C2	0.0414 (12)	0.0268 (10)	0.0333 (12)	0.0016 (9)	−0.0040 (9)	−0.0017 (9)
C3	0.0533 (14)	0.0280 (11)	0.0354 (13)	−0.0024 (10)	−0.0111 (10)	0.0007 (9)
C4	0.0427 (12)	0.0288 (11)	0.0336 (12)	0.0000 (9)	−0.0074 (9)	−0.0031 (9)
C5	0.0497 (14)	0.0275 (11)	0.0394 (13)	−0.0007 (10)	−0.0042 (10)	−0.0029 (10)
C6	0.127 (6)	0.053 (3)	0.049 (3)	−0.053 (3)	−0.024 (4)	0.005 (2)
C7	0.049 (3)	0.033 (2)	0.042 (3)	0.000	−0.020 (2)	0.000
C8	0.0486 (14)	0.0405 (13)	0.0402 (14)	0.0048 (11)	−0.0044 (10)	0.0007 (11)
C9	0.0528 (16)	0.0609 (18)	0.0456 (15)	0.0178 (13)	−0.0006 (12)	0.0010 (13)
C10	0.043 (2)	0.084 (3)	0.042 (2)	0.000	−0.0003 (16)	0.000
C11	0.0655 (18)	0.0355 (14)	0.069 (2)	−0.0091 (12)	0.0129 (15)	−0.0069 (13)
C12	0.0578 (16)	0.0388 (13)	0.0433 (14)	−0.0068 (11)	0.0000 (11)	−0.0039 (11)
C13	0.0384 (12)	0.0330 (12)	0.0419 (13)	−0.0018 (9)	−0.0019 (10)	0.0015 (10)
C14	0.076 (2)	0.0456 (15)	0.0580 (18)	−0.0175 (14)	−0.0252 (15)	0.0147 (13)
C15	0.083 (2)	0.0592 (19)	0.061 (2)	−0.0065 (16)	−0.0203 (16)	0.0207 (16)

### Geometric parameters (Å, º)

**Table:**

Co1—O5	2.076 (2)	C6—H6A	0.9700
Co1—N3	2.138 (3)	C6—H6B	0.9700
Co1—O1	2.1441 (15)	C7—C6^i^	1.557 (6)
Co1—O1^i^	2.1441 (15)	C7—H7A	0.9700
Co1—N1^i^	2.1515 (17)	C7—H7B	0.9700
Co1—N1	2.1515 (17)	C8—C9	1.378 (4)
O1—C4	1.249 (3)	C8—H8	0.9300
O2—C4	1.264 (3)	C9—C10	1.369 (3)
O3—C5	1.297 (3)	C9—H9	0.9300
O3—H3	0.8200	C10—C9^i^	1.369 (3)
O4—C5	1.215 (3)	C10—H10	0.9300
O5—H1W	0.8200	C11—C12	1.387 (4)
N1—C3	1.326 (3)	C11—H11	0.9300
N1—C1	1.374 (3)	C12—C13	1.378 (3)
N2—C3	1.348 (3)	C12—H12	0.9300
N2—C2	1.366 (3)	C13—C14	1.390 (3)
N2—H2	0.8600	C13—C13^ii^	1.486 (4)
N3—C8	1.340 (3)	C14—C15	1.362 (4)
N3—C8^i^	1.340 (3)	C14—H14	0.9300
N4—C15	1.322 (4)	C15—H15	0.9300
N4—C11	1.327 (4)	C7'—C6'^i^	1.667 (13)
C1—C2	1.367 (3)	C7'—C6'	1.667 (13)
C1—C4	1.487 (3)	C7'—H7'1	0.9700
C2—C5	1.482 (3)	C7'—H7'2	0.9700
C3—C6	1.478 (6)	C6'—H6'1	0.9700
C3—C6'	1.565 (19)	C6'—H6'2	0.9700
C6—C7	1.557 (6)		
			
O5—Co1—N3	176.35 (10)	C3—C6—H6A	108.6
O5—Co1—O1	87.35 (7)	C7—C6—H6A	108.6
N3—Co1—O1	90.22 (7)	C3—C6—H6B	108.6
O5—Co1—O1^i^	87.35 (7)	C7—C6—H6B	108.6
N3—Co1—O1^i^	90.22 (7)	H6A—C6—H6B	107.6
O1—Co1—O1^i^	96.30 (8)	C6—C7—C6^i^	110.7 (7)
O5—Co1—N1^i^	87.43 (7)	C6—C7—H7A	109.5
N3—Co1—N1^i^	94.74 (7)	C6^i^—C7—H7A	109.5
O1—Co1—N1^i^	172.62 (6)	C6—C7—H7B	109.5
O1^i^—Co1—N1^i^	78.25 (6)	C6^i^—C7—H7B	109.5
O5—Co1—N1	87.43 (7)	H7A—C7—H7B	108.1
N3—Co1—N1	94.74 (7)	N3—C8—C9	123.0 (2)
O1—Co1—N1	78.25 (6)	N3—C8—H8	118.5
O1^i^—Co1—N1	172.62 (6)	C9—C8—H8	118.5
N1^i^—Co1—N1	106.72 (9)	C10—C9—C8	119.2 (3)
C4—O1—Co1	115.09 (14)	C10—C9—H9	120.4
C5—O3—H3	109.5	C8—C9—H9	120.4
Co1—O5—H1W	109.5	C9—C10—C9^i^	118.7 (4)
C3—N1—C1	105.87 (18)	C9—C10—H10	120.7
C3—N1—Co1	143.14 (15)	C9^i^—C10—H10	120.7
C1—N1—Co1	109.78 (13)	N4—C11—C12	124.3 (3)
C3—N2—C2	108.63 (18)	N4—C11—H11	117.8
C3—N2—H2	125.7	C12—C11—H11	117.8
C2—N2—H2	125.7	C13—C12—C11	118.8 (3)
C8—N3—C8^i^	116.9 (3)	C13—C12—H12	120.6
C8—N3—Co1	121.51 (15)	C11—C12—H12	120.6
C8^i^—N3—Co1	121.51 (15)	C12—C13—C14	116.7 (2)
C15—N4—C11	116.1 (2)	C12—C13—C13^ii^	122.5 (3)
C2—C1—N1	110.02 (19)	C14—C13—C13^ii^	120.9 (3)
C2—C1—C4	131.70 (19)	C15—C14—C13	120.0 (3)
N1—C1—C4	118.24 (18)	C15—C14—H14	120.0
N2—C2—C1	105.15 (18)	C13—C14—H14	120.0
N2—C2—C5	121.5 (2)	N4—C15—C14	124.1 (3)
C1—C2—C5	133.3 (2)	N4—C15—H15	118.0
N1—C3—N2	110.33 (19)	C14—C15—H15	118.0
N1—C3—C6	129.0 (3)	C6'^i^—C7'—C6'	93.5 (12)
N2—C3—C6	120.4 (3)	C6'^i^—C7'—H7'1	113.0
N1—C3—C6'	123.2 (7)	C6'—C7'—H7'1	113.0
N2—C3—C6'	125.5 (6)	C6'^i^—C7'—H7'2	113.0
C6—C3—C6'	14.6 (7)	C6'—C7'—H7'2	113.0
O1—C4—O2	125.2 (2)	H7'1—C7'—H7'2	110.4
O1—C4—C1	117.23 (18)	C3—C6'—C7'	101.5 (9)
O2—C4—C1	117.59 (19)	C3—C6'—H6'1	111.5
O4—C5—O3	122.8 (2)	C7'—C6'—H6'1	111.5
O4—C5—C2	121.0 (2)	C3—C6'—H6'2	111.5
O3—C5—C2	116.2 (2)	C7'—C6'—H6'2	111.5
C3—C6—C7	114.5 (5)	H6'1—C6'—H6'2	109.3
			
O5—Co1—O1—C4	77.25 (17)	C1—N1—C3—C6	174.5 (5)
N3—Co1—O1—C4	−105.47 (17)	Co1—N1—C3—C6	9.5 (6)
O1^i^—Co1—O1—C4	164.28 (13)	C1—N1—C3—C6'	−169.0 (6)
N1^i^—Co1—O1—C4	122.3 (5)	Co1—N1—C3—C6'	26.1 (7)
N1—Co1—O1—C4	−10.68 (16)	C2—N2—C3—N1	−0.1 (3)
O5—Co1—N1—C3	86.0 (3)	C2—N2—C3—C6	−174.9 (4)
N3—Co1—N1—C3	−96.9 (3)	C2—N2—C3—C6'	168.9 (7)
O1—Co1—N1—C3	173.8 (3)	Co1—O1—C4—O2	−169.8 (2)
O1^i^—Co1—N1—C3	131.0 (5)	Co1—O1—C4—C1	9.7 (3)
N1^i^—Co1—N1—C3	−0.5 (3)	C2—C1—C4—O1	−178.4 (2)
O5—Co1—N1—C1	−78.58 (15)	N1—C1—C4—O1	−1.1 (3)
N3—Co1—N1—C1	98.49 (15)	C2—C1—C4—O2	1.1 (4)
O1—Co1—N1—C1	9.25 (14)	N1—C1—C4—O2	178.5 (2)
O1^i^—Co1—N1—C1	−33.6 (6)	N2—C2—C5—O4	−0.9 (4)
N1^i^—Co1—N1—C1	−165.12 (11)	C1—C2—C5—O4	176.1 (2)
O5—Co1—N3—C8	91.7 (2)	N2—C2—C5—O3	180.0 (2)
O1—Co1—N3—C8	43.6 (2)	C1—C2—C5—O3	−3.1 (4)
O1^i^—Co1—N3—C8	139.9 (2)	N1—C3—C6—C7	27.9 (8)
N1^i^—Co1—N3—C8	−141.9 (2)	N2—C3—C6—C7	−158.5 (4)
N1—Co1—N3—C8	−34.6 (2)	C6'—C3—C6—C7	−44 (3)
O5—Co1—N3—C8^i^	−91.7 (2)	C3—C6—C7—C6^i^	−114.7 (5)
O1—Co1—N3—C8^i^	−139.9 (2)	C8^i^—N3—C8—C9	−1.4 (5)
O1^i^—Co1—N3—C8^i^	−43.6 (2)	Co1—N3—C8—C9	175.34 (19)
N1^i^—Co1—N3—C8^i^	34.6 (2)	N3—C8—C9—C10	0.1 (4)
N1—Co1—N3—C8^i^	141.9 (2)	C8—C9—C10—C9^i^	1.3 (5)
C3—N1—C1—C2	−0.4 (3)	C15—N4—C11—C12	0.6 (4)
Co1—N1—C1—C2	170.09 (15)	N4—C11—C12—C13	−0.2 (4)
C3—N1—C1—C4	−178.2 (2)	C11—C12—C13—C14	−0.2 (4)
Co1—N1—C1—C4	−7.8 (2)	C11—C12—C13—C13^ii^	179.1 (3)
C3—N2—C2—C1	−0.1 (3)	C12—C13—C14—C15	0.3 (4)
C3—N2—C2—C5	177.6 (2)	C13^ii^—C13—C14—C15	−179.0 (3)
N1—C1—C2—N2	0.3 (2)	C11—N4—C15—C14	−0.4 (5)
C4—C1—C2—N2	177.8 (2)	C13—C14—C15—N4	0.0 (5)
N1—C1—C2—C5	−177.0 (2)	N1—C3—C6'—C7'	−70.7 (10)
C4—C1—C2—C5	0.5 (4)	N2—C3—C6'—C7'	121.7 (6)
C1—N1—C3—N2	0.3 (3)	C6—C3—C6'—C7'	48 (2)
Co1—N1—C3—N2	−164.64 (19)	C6'^i^—C7'—C6'—C3	129.0 (6)

Symmetry codes: (i) *x*, −*y*+1/2, *z*; (ii) −*x*+1, −*y*+1, −*z*+2.

### Hydrogen-bond geometry (Å, º)

**Table:**

*D*—H···*A*	*D*—H	H···*A*	*D*···*A*	*D*—H···*A*
O3—H3···O2	0.82	1.65	2.473 (2)	177
O5—H1*W*···N4^iii^	0.82	1.95	2.727 (3)	157
N2—H2···O4^iv^	0.86	1.93	2.763 (3)	162

Symmetry codes: (iii) −*x*+2, *y*−1/2, −*z*+1; (iv) −*x*+2, −*y*+1, −*z*.

## References

[bb1] Bruker (2001). *SMART* and *SAINT* Bruker AXS Inc., Madison, Wisconsin, USA.

[bb2] Feng, X., Zhao, J. S., Liu, B., Wang, L. Y., Ng, S., Zhang, G., Wang, J. G., Shi, X. G. & Liu, Y. Y. (2010). *Cryst. Growth Des.* **10**, 1399–1408.

[bb3] Li, X., Wu, B. L., Niu, C. Y., Niu, Y. Y. & Zhang, H. Y. (2009). *Cryst. Growth Des.* **9**, 3423–3431.

[bb4] Li, X., Wu, B. L., Wang, R. Y., Zhang, H. Y., Niu, C. Y., Niu, Y. Y. & Hou, H. W. (2010). *Inorg. Chem.* **49**, 2600–2613. 10.1021/ic901113p 20141153

[bb5] Liu, X. F., Wang, L. Y., Ma, L. F. & Li, R. F. (2010). *Chin. J. Struct. Chem.* **29**, 280–284.

[bb6] Lu, W. G., Jiang, L. & Lu, T. B. (2010). *Cryst. Growth Des.* **10**, 4310–4318.

[bb7] Sheldrick, G. M. (2001). *SADABS* University of Göttingen, Germany.

[bb8] Sheldrick, G. M. (2008). *Acta Cryst.* A**64**, 112–122. 10.1107/S0108767307043930 18156677

[bb9] Song, J. F., Zhou, R. S., Hu, T. P., Zhuo, C. & Wang, B. B. (2010). *J. Coord. Chem.* **63**, 4201–4214.

[bb10] Wang, S., Zhang, L. R., Li, G. H., Huo, Q. S. & Liu, Y. L. (2008). *CrystEngComm*, **10**, 1662–1666.

[bb11] Zhang, F. W., Li, Z. F., Ge, T. Z., Yao, H. C., Li, G., Lu, H. J. & Zhu, Y. Y. (2010). *Inorg. Chem.* **49**, 3776–3788. 10.1021/ic902483m 20230024

[bb12] Zheng, S. R., Cai, S. L., Pan, M., Fan, J., Xiao, T. T. & Zhang, W. G. (2011). *CrystEngComm*, **13**, 883–888.

[bb13] Zhu, Y., Wang, W. Y., Guo, M. W., Li, G. & Lu, H. J. (2011). *Inorg. Chem. Commun.* **14**, 1432–1435.

[bb14] Zhu, L. C., Zhao, Y., Yu, S. J. & Zhao, M. M. (2010). *Inorg. Chem. Commun.* **13**, 1299–1303.

